# High-fat diet-induced obesity affects alpha 7 nicotine acetylcholine receptor expressions in mouse lung myeloid cells

**DOI:** 10.1038/s41598-020-75414-w

**Published:** 2020-10-27

**Authors:** Yong Qi, Dan Si, Li Zhu, Yanan Qi, Zhuhua Wu, Dan Chen, Yunlei Yang

**Affiliations:** 1 Department of Pulmonary and Critical Care Medicine, People’s Hospital of Zhengzhou University, Henan Provincial People’s Hospital, Zhengzhou, 450003 Henan China; 2 Department of Respiratory Medicine of Central China Fuwai Hospital, Central China Fuwai Hospital of Zhengzhou University, Zhengzhou, 450003 Henan China; 3 Department of Medicine Division of Endocrinology, Albert Einstein College of Medicine, Bronx, New York, USA; 4 Department of Neuroscience, Albert Einstein College of Medicine, Bronx, New York, USA; 5 Einstein-Mount Sinai Diabetes Research Center, Albert Einstein College of Medicine, Bronx, New York, USA; 6 The Fleischer Institute for Diabetes and Metabolism, Albert Einstein College of Medicine, Bronx, New York, USA

**Keywords:** Cell biology, Cell biology, Cell biology, Medical research, Medical research

## Abstract

Ample evidence indicates that obesity causes dysfunctions in the lung. Previous studies also show that cholinergic anti-inflammatory pathways play crucial roles in obesity-induced chronic inflammation via α7 nicotinic acetylcholine receptor (α7nAChR) signaling. However, it remains unclear whether and how obesity affects the expressions of α7nAChR in myeloid cells in the lung. To address this question, we treated regular chow diet-fed mice or high-fat diet induced obese mice with lipopolysaccharide (LPS) or vehicle via endotracheal injections. By using a multicolor flow cytometry approach to analyze and characterize differential cell subpopulations and α7nAChR expressions, we find no detectable α7nAChR in granulocytes, monocytes and alveolar macrophages, and low expression levels of α7nAChR were detected in interstitial macrophages. Interestingly, we find that a challenge with LPS treatment significantly increased expression levels of α7nAChR in monocytes, alveolar and interstitial macrophages. Meanwhile, we observed that the expression levels of α7nAChR in alveolar and interstitial macrophages in high-fat diet induced obese mice were lower than regular chow diet-fed mice challenged by the LPS. Together, our findings indicate that obesity alters the expressions of α7nAChR in differential lung myeloid cells.

## Introduction

The rates of obesity have been increasing worldwide^1^. According to epidemiological data, obesity is associated with adverse outcomes and increased morbidity^2^ including dysfunction in the lung disorders^3^. Previous studies also show that obesity increases the rates of mortality in conditions of aspiration pneumonia, bronchial asthma, chronic obstructive pulmonary disease, obstructive sleep apnea syndrome, acute lung injury and ARDS (acute/ adult respiratory distress syndrome)^4–9^. Emerging evidence also indicates that obesity may alter lung immune functions^10,11^. However, the involved molecular and cellular mechanisms remain unclear.

It is clear that obesity induces low-grade chronic inflammation (hereafter referred to as “metaflammation”) by increasing the release of pro-inflammatory cytokines, including tumor necrosis factor-α (TNF-α), leptin, and adipose tissue-derived resistin. Contrastingly, obesity reduces the levels of anti-inflammatory adipokines (i.e., adiponectin, omentin, SFRP5, vaspin, ZAG, and IL-10)^12^. Interestingly, low-grade chronic inflammation was predominantly involved in lung dysfunctions related to obesity. There is evidence to show that leukocytes in lung were increased in diet-induced obese (DIO) mice^11^. Monocyte chemotactic protein-1 (MCP-1) and TNF-α were also detected in lung tissue and bronchoalveolar lavage fluid^13,14^. Meanwhile, lipopolysaccharide (LPS) treatment induces changes in inflammatory-related gene expressions, and approximately half of the genes induced by LPS-stimulated lung inflammation were specific in DIO mice^15^. These results suggest that the metabolic phenotypes of obesity probably partake in the LPS-induced inflammation in lungs. Neuroimmune systems play an important role in the control of inflammation levels as the nervous and immune system interact with each other^16^. The cholinergic anti-inflammatory pathway (CAP) inhibits inflammation by suppressing cytokine synthesis via the release of acetylcholine (ACh) in reticuloendothelial systems including lung, spleen, liver, kidneys and gastrointestinal tract. Activation of α7nAChR in macrophages and other cytokine-producing cells inhibits proinflammatory cytokine synthesis, which is probably attributable to α7nAChR signaling in macrophages-induced suppression of TNF-α release and other proinflammatory cytokines pathway^17–21^. Previous studies demonstrate that several lung cell populations express α7nAChR receptors^22–26^ and α7nAChR are implicated in lung inflammation^21,25–27^. For example, intra-tracheal administration of α7nAChR agonist 3-(2,4-dimethoxybenzylidene)-anabasine (GTS-21)^21,25,26^ reduced the inflammatory cytokines in the lung, and N-(3R)-1-azabicyclo[2,2,2]oct-3-yl-(4-chlorobenzamide) (PNU-282987, a specific agonist for α7nAChR) reduced alveolar exudation in acid-induced injured lungs. However, the mechanism by which the α7nAChR regulates obesity-induced lung inflammation remains unclear.

Rapid upregulation of α7nAChR by tyrosine dephosphorylation and co-expression of the gene hRIC3 (resistant to inhibitors of cholinesterase) produces a two-fold increase in α7 receptor function^28,29^. Previous gene chip data in the GEO database^15^ showed no apparent difference in total α7nAChR mRNA transcription levels of the lungs between regular weight and DIO mice; however, these studies did not analyze the changes in α7nAChR expressions in single-cell populations. We therefore set off to identify the lung cell populations expressing α7nAChR and define the associated alterations in high-fat diet-induced obese mice with and without LPS treatment. By applying multicolor flow cytometry approach, in this study we identified α7nAChR expressing populations, and determined the proportion of each population and α7nAChR alterations in obese lungs with or without LPS treatment.

## Methods

### Reagents

PE-CF594 rat anti-mouse CD24 (562477, 2.5:100), BB515 rat anti-mouse I-A/I-E (MHCII 565254, 1.25:100) and PE rat anti-mouse IgG1 κ isotype control (554685, 5:100) were purchased from the BD Bioscience company. PerCP-Cy5.5 rat anti-mouse CD45 (45-0451-82, 1:100), APC rat anti-mouse IgG2b κ isotype control (17-4031-82, 1.25:100) and APC rat anti-mouse CD206 (17-2061-82, 1.25:100) were purchased from eBioscience. The PE rat anti-mouse α7AChR (sc-58607PE, 5:100) was purchased from Santa Cruz. APC-Cy7 rat anti-mouse CD11b (101226, 2.5:100), PE-Cy7 hamster anti-mouse CD11c (117318, 1.25:100) , Brilliant Violet 421 rat anti-mouse CD86 (105032, 1:100) , Brilliant Violet 421 rat IgG2a κ isotype control (400536, 1:100) , APC rat anti-mouse CD24 (138506, 3.75:100) and Brilliant Violet 421 mouse anti-mouse CD64 (139309, 1:100) were purchased from the Biolegend. Lipid droplet dye, BODIPY™ 493/503 (D-3922, 1:200) was purchased from the Invitrogen. Rabbit anti-mouse CD68 antibody (BA3638, 1:100) was purchased from Boster. Rabbit anti-mouse F4/80 antibody (GB11027, 1:800) was purchased from Servicebio.

### Animal treatment

Six-week-old male C57BL/6 J mice were purchased from Beijing Vital River Laboratory Animal Technology Co. Ltd. Mice were housed in climate-controlled areas (20–24 °C, 40–50% humidity, 12 h light/12 h dark cycle) and randomly divided into a regular chow diet (irradiated experimental pellet feed, Experimental Animal Center of Henan Province, 11% kcal fat) group (RCD) and a high-fat diet (Research Diets, D12492, 60% kcal fat) group (HFD). After 18 weeks, according to the weight standards of previous reports^30,31^, the mice were classified as obesity when their body weight were 20% more than the average weight of the RCD group; mice whose body weight did not reach the obesity standard in the HFD group and the body weight reached the obesity standard in the RCD group were excluded. (see Supplementary Fig. S1 online). All experiments were approved by the Ethics committee of the experimental animals of Zhengzhou University and the protocol was strictly aligned to the ethical guidelines of the experimental animals of Zhengzhou University.

### Glucose tolerance test

Glucose tolerance test (GTT) was performed following 18 weeks of feeding with high-fat diet or regular chow diet in the two groups. The mice were fasted for 16 h^32^ and subsequently received intraperitoneal injections (i.p.) of glucose at a dose of 2 g/kg of body weight. The blood samples (25–50 μl) were collected from the tail of the mice immediately after the injection at 0, 30, 60, 90 and 120 min, respectively to determine the blood glucose levels. The procedure was performed by an Accu-Chek Performa II instrument (Roche, USA).

### Induction of lung inflammation in mice

Direct endotracheal intubation techniques were used for intratracheal LPS instillation to establish a mouse model of acute lung inflammation^25,33,34^. Mice were anesthetized with pentobarbital, and suspended on a customized operating platform after anesthesia as a previously described method^34^. Mouse tongue was pulled out by a dental instrument to see the light leaking from the glottis of the mouse. When the glottis was open, a 22G venous indwelling needle cannula was inserted into the trachea, and 100 μg LPS (O55:B5, L2880, Sigma) with 30 μl saline was instilled in LPS treated groups. The control groups received the same dose of saline. Mice were finally divided into four groups (HFD + LPS, n = 10; HFD + vehicle, n = 8; RCD + LPS, n = 8; RCD + vehicle, n = 8). No mice that underwent the above procedure were dead before harvest. After 24 h of LPS or saline treatment, the mice were sacrificed and their lung tissues were harvested for subsequent procedures (see Supplementary Fig. S1 online).

### Lung histology

Lung tissues were sampled and fixed in 4% paraformaldehyde for 72 h. Then sample was embedded in paraffin and 4 μm thick sections were prepared for sectioning. Then the lung tissues were stained with hematoxylin and eosin (HE staining) as previous protocol. For lung Oil red O staining, lung tissues were snap frozen in liquid nitrogen, embedded in OCT, and prepared fresh frozen sections. Then the lung sections were fixed by Universal Tissue Fixative and washed with distilled water, dried and stained with 0.3% Oil red O solution for 15 min. After washed and color separated, the sections were re-stained with hematoxylin for 1 min, rinsed with distilled water and subsequently washed with a glycerin-based sealant. All images were obtained using Pannoramic DESK section scan system (3D HISTECH, Hungary).

### Immunofluorescence staining

Fresh frozen lung sections were rewarmed and slightly dried, and then incubated with BSA for 30 min to achieve the block effect, and then incubated with the Bodipy dye for 1 h. Then the sections were added with CD68 or F4/80 primary antibody and incubated overnight. Afterwards, washed 3 times with PBS and slightly dried, the sections were incubated with a secondary antibody for 50 min. Washed and dried again, then the sections were incubated with DAPI for 10 min. Finally, the lung sections were mounted with anti-fluorescent attenuation medium after the sections were rinsed with PBS. All images were obtained using Pannoramic DESK section scan system (3D HISTECH, Hungary).

To measure fluorescence signal intensity of the lipid deposition in macrophages in RCD and HFD mice with or without LPS treatment respectively, an image analysis method based on ImageJ software was used to quantify differences in the signal intensity of lipid particles in macrophages. Briefly, 10 images of field with 40 × magnification were randomly captured from each of the slice, then the images were first thresholded to eliminate background fluorescence. Selection zones were established by CD68^+^ or F4/80^+^ macrophages in each image. CD68^+^ or F4/80^+^ macrophages (n = 240 cells/12 slices/4 mice) were selected from each group and subsequently analyzed for the signal intensity of Bodipy-labeled lipid particles in selected macrophages. We normalized the signal intensity of Bodipy from average RCD mice for each group.

### Flow cytometry analysis of lung myeloid population

Lung single-cell suspension was prepared according to previous protocol^35^. The single-cell suspension was diluted to 1 × 10^6^ cells/100 μl with stain buffer in a flow cytometry stain tube. Then mixed antibody cocktails were carefully added to the lung single-cell suspension and vortexed to prepare the experimental groups, meanwhile, the isotype controls and negative controls were also prepared similarly. For intracellular stain of CD206, permeabilization was performed according to manufactures instruction. All procedures were performed in accordance with the product instructions. Each group of samples was analyzed with a FACS Aria flow cytometer (BD Company). We can collect around 100 α7nAChR^+^ cells without LPS challenge and around 2000 α7nAChR^+^ cells after LPS challenge for each sample by the method described above. Then the raw data were analyzed offline by the FlowJo v10 software.

### Statistical analysis

The results were expressed as mean ± SD. Normality test was determined by Shapiro–Wilk. If the data wasn’t fit in normal distribution, then logarithmic transformation was performed. For repeated measured data such as body weight and blood glucose level, the ANOVA for repeated measured data were performed. For data with homogeneous variances, the post-hoc tests were done by LSD-t method; For data with heterogeneous variances, logarithmic transformation was performed. If the variances were homogeneous after the procedure, then the post-hoc tests were done by LSD-t method as above. If the variances were still heterogeneous, Welch method was used and the post-hoc tests were done by Games–Howell method. Required sample size was calculated via SPSS and G*Power software post hoc, the detailed calculation process was in supplemental materials. The differences were considered statistically significant if the P-value was lower than 0.05 (*P* < 0.05).

## Results

### Obesity-induced metabolic and morphological changes in Mouse Lungs

**Figure 1:**
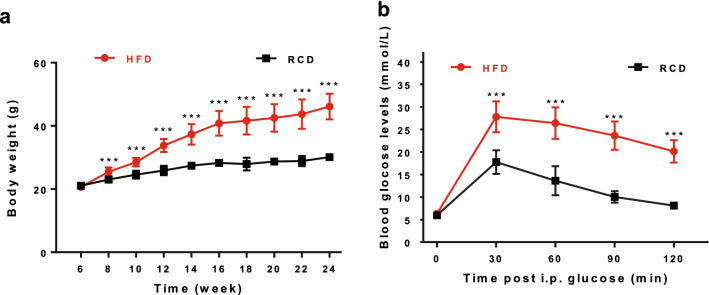
Development of obese mice phenotypes. (**a**) Body weight of the mice fed with high-fat diet (filled circles in red, n = 18) was significantly increased, in comparison to control mice fed with regular chow diet (filled circles in black, n = 16). (**b**) Fast blood glucose level and glucose tolerance were impaired in HFD mice (filled circles in red, n = 18) as compared to RCD mice (filled circles in black, n = 16). Detailed data can be seen in Supplementary Table S4, S5. ***P < 0.001 by ANOVA for repeated measured data, post-hoc Bonferroni method.

Mice fed a high-fat diet showed greater body weight gains than those fed regular chow (Fig. 1a). Glucose tolerance tests (GTTs) were subsequently performed, and we observed that the blood glucose levels of HFD group mice were higher than that of RCD group mice at 30, 60, 90 and 120 min post intraperitoneal (i.p.) injections of glucose (Fig. 1b), indicating metabolic phenotypes of diet-induced obesity.

**Figure 2:**
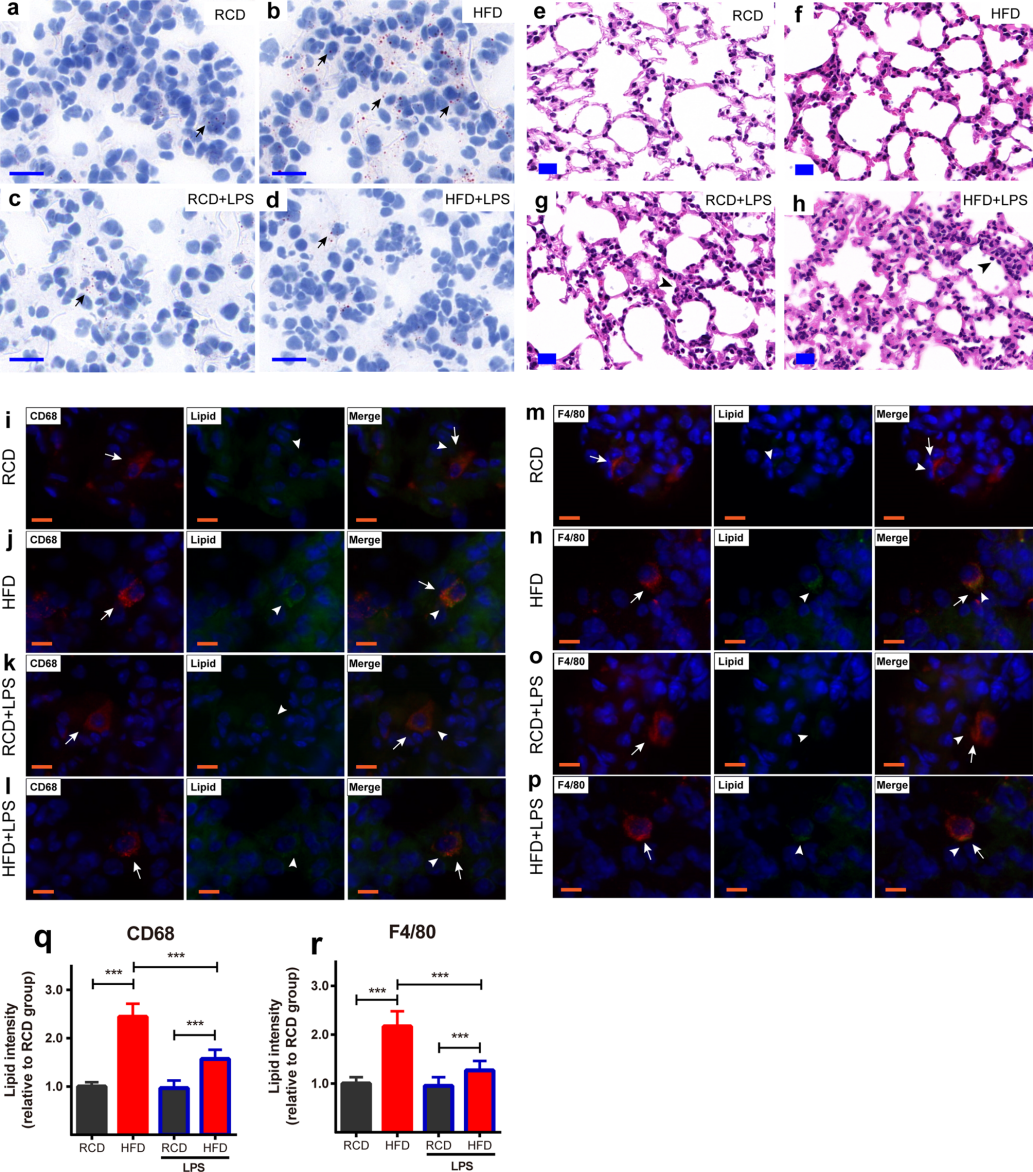
Obesity-induced pathological changes in lungs. (**a–d**) Oil Red O staining of lung in RCD and HFD mice. Less fats accumulated in the lung tissue (arrows pointing) in RCD mice (**a**), RCD + LPS mice (**c**). A large quantity of lipid droplets (arrows pointing) were accumulated in lung tissue in HFD mice (**b**) and less fat deposits in HFD + LPS mice (**d**). (**a**–**d**) scale bars represent 20 μm, the nuclei were re-stained blue with hematoxylin. (**e–h**) HE staining of lungs in RCD and HFD mice. HE staining of RCD mice 24 h after intratracheal administration of saline (**e**). HE staining of HFD mice 24 h after intratracheal administration of saline (**f**). HE staining of RCD mice 24 h after intratracheal administration of LPS (**g**). HE staining of HFD mice 24 h after intratracheal administration of LPS, arrows designate the alveolar inflammatory infiltration and alveolar wall thickening after LPS challenge (**h**). (**e–h**) scale bars represent 20 μm. (**i–l**) Lung immunofluorescence staining of CD68^+^ macrophages in RCD and HFD mice. CD68^+^ macrophages were stained red (arrow pointing). Lipid droplets were stained green (arrow head pointing) by Bodipy. Representative image of CD68^+^ macrophages observed in HFD (**j**) mice, the merged images showed the lipid droplets were located in the macrophages of the HFD mice (**j**) and the lipid droplets decreased in macrophages of HFD + LPS (**l**) mice. Representative images of CD68^+^ macrophages observed in RCD (**i**) and RCD + LPS (**k**) mice, the merged images indicated there were less lipid droplets in macrophages. (**i–l**) scale bars represent 10 μm. (**m–p**) Lung immunofluorescence staining of F4/80^+^ macrophages in RCD and HFD mice. F4/80^+^ macrophages were stained red (arrow pointing). Lipid droplets were stained green (arrow head pointing) by Bodipy. Representative image of F4/80^+^ macrophages were observed in HFD (**n**) mice, the merged images showed the lipid droplets located in the F4/80^+^ macrophages in HFD (**n**) mice and the lipid droplets decreased in macrophages of HFD + LPS (**p**) mice. Representative images of F4/80^+^ macrophages were observed in RCD (**m**), RCD + LPS (**o**) mice, the merged images indicated there were less lipid droplets in F4/80^+^ macrophages. (**m–p)** scale bars represent 10 μm. (**q–r**) Quantification analysis of lipid accumulation in each group (n = 240 cells/12 slices/4 mice for each group). Data were normalized to the averaged signal intensity from RCD mice for each group. (**q**) Lipid intensity in CD68^+^ macrophages, RCD: 1.0 ± 0.09; HFD: 2.44 ± 0.27; RCD + LPS: 0.97 ± 0.16; HFD + LPS: 1.57 ± 0.19. (**r**) Lipid intensity in F4/80^+^ macrophages, RCD: 1.0 ± 0.13; HFD: 2.17 ± 0.31; RCD + LPS: 0.95 ± 0.18; HFD + LPS: 1.27 ± 0.19. ***P < 0.001, significant differences between each group (ANOVA, LSD-t post hoc).

**Figure 3:**
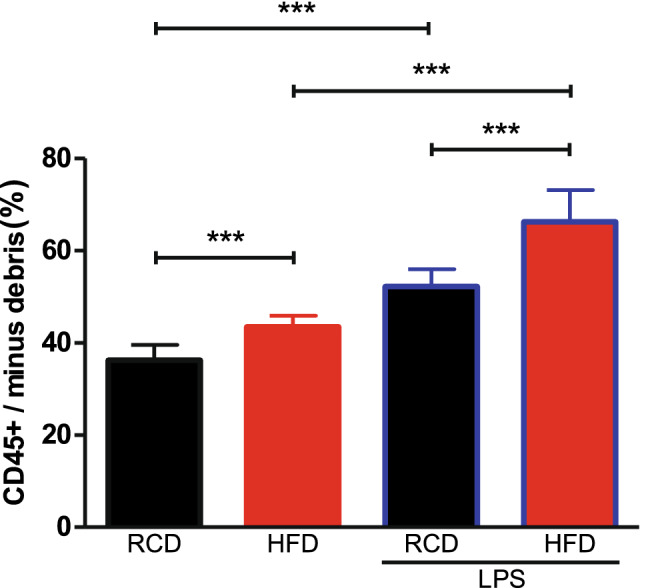
Identification of myeloid cell populations in mouse lung tissues. The proportion of leukocyte (CD45^+^) was increased after stimulation of LPS. HFD mice have more CD45^+^ cells than RCD mice. Detailed statistical results can be seen in Supplementary Table S6. RCD group, n = 8; RCD + LPS group, n = 8; HFD group, n = 8; HFD + LPS group, n = 10. *P* < 0.001, HFD group comparing to RCD group; *P* < 0.001, RCD + LPS group comparing to RCD group; *P* < 0.001, HFD + LPS group comparing to HFD group; *P* < 0.001, HFD + LPS group comparing to RCD + LPS group. RCD, regular chow diet. HFD, high-fat diet. LPS, lipopolysaccharide. Data were presented as Mean ± SD. ***P < 0.001 by ANOVA after logarithmic transformation, post-hoc LSD-t method.

Oil red O staining of animal lung tissue showed that HFD mice had more lipid deposits than RCD mice in the lungs (Fig. 2a,b). LPS challenge significantly reduced the deposition of lipid droplets in the lungs collected from HFD mice (Fig. 2d). Further immunofluorescence staining indicated that part of the lipid particles existed in CD68^+^ macrophages and F4/80^+^ macrophages (Fig. 2i–p). Quantification analysis showed that more lipid particles accumulated in CD68^+^ and F4/80^+^ macrophages in HFD mice than RCD mice with or without LPS challenge (Fig. 2q,r), and the lipid particles decreased in CD68^+^ macrophages and F4/80^+^ macrophages in HFD mice after LPS challenge (Fig. 2q,r). There were few lipid particles in macrophages of RCD mice and no significant decrease after LPS stimulation. H&E staining on lung sections showed no obvious inflammatory infiltration in RCD (Fig. 2e) and HFD (Fig. 2f) mice without LPS treatment; however, LPS treatment induced inflammatory infiltration and thickening of alveolar walls in both RCD (Fig. 2g) and HFD mice (Fig. 2h). Consistently, our flow cytometry results showed that LPS treatment increased the amount of lung inflammatory exudation more in HFD mice than RCD mice (Fig. 3).

### Identification of myeloid cell populations in mouse lung tissues

We next sought to define the myeloid cell populations in the inflammatory lung by utilizing a gating strategy modified according to published protocols^36^ (see Supplementary Fig. S2 online). The leukocytes were identified by using the surface marker CD45. In the CD45^+^ cell population, alveolar macrophages were identified as CD11b^-^CD11c^+^CD64^+^CD24^-^ cells. Since nearly all CD11b^−^CD11c^+^CD24^−^ cells were CD64^+^, alveolar macrophages were identified as CD11b^−^CD11c^+^CD24^−^ in our simplified gating strategy. Monocytes were identified as CD11b^+^CD11c^+/−^CD64^low^MHC-II^−^CD24^low^ or CD11b^+^CD11c^+/−^MHC-II^−^CD24^low^. Granulocytes and interstitial macrophages were identified following previously published protocols^36^.

### Differential changes of cell populations in obesity and LPS treatment

**Figure 4:**
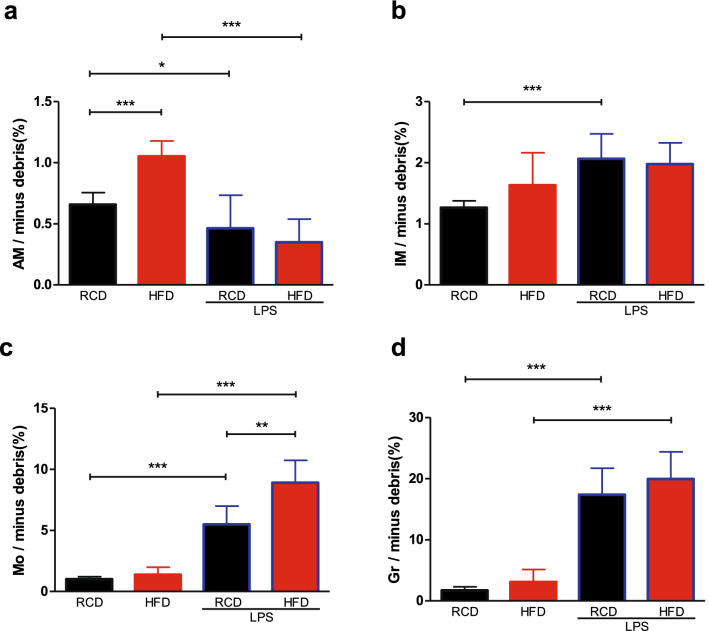
Differential changes of cell populations in obesity and LPS treatment. The percentage of interstitial macrophages (**b**), granulocytes (**d**) and monocytes (**c**) were intensified after stimulation of LPS and the percentage of alveolar macrophages (**a**) was diminished. High-fat diet-induced obesity had an impact on the number of alveolar macrophages and monocytes. Detailed statistical results can be seen in Supplementary Table S6. RCD group, n = 8; RCD + LPS group, n = 8; HFD group, n = 8; HFD + LPS group, n = 10. Alveolar macrophages/minus debris (%): by ANOVA, post-hoc LSD-t method. *P* < 0.001, comparing HFD group to RCD group; *P* < 0.05, comparing RCD + LPS group to RCD group; *P* < 0.001, comparing HFD + LPS group to HFD group. Interstitial macrophages/minus debris (%): by ANOVA after logarithmic transformation, post-hoc LSD-t method. *P* < 0.001, comparing RCD + LPS group to RCD group. Monocytes/minus debris (%): by ANOVA after logarithmic transformation, post-hoc LSD-t method. *P* < 0.001, comparing RCD + LPS group to RCD group; *P* < 0.001, comparing HFD + LPS group to HFD group; *P* < 0.01, comparing HFD + LPS group to RCD + LPS group. Granulocytes/minus debris (%): by Welch’s ANOVA, post-hoc Games–Howell method. *P* < 0.001, comparing RCD + LPS group to RCD group. *P* < 0.001, comparing HFD + LPS group to HFD group. RCD, regular chow diet. HFD, high-fat diet. LPS, lipopolysaccharide. IM, interstitial macrophages. Mo, monocytes. AM, alveolar macrophages. Gr, Granulocytes. Data were presented as Mean ± SD. ***P < 0.001; **P < 0.01; *P < 0.05.

We noticed that there were more CD45^+^ cells in HFD mice lungs than RCD mice with or without LPS challenge (Fig. 3). The percentages of alveolar macrophages in HFD mice were higher than RCD mice (Fig. 4a). Similarly, both monocytes and granulocytes were increased following LPS stimulation (Fig. 4c,d). Further investigation showed that more monocytes were recruited in HFD mice than RCD mice, and LPS treatment decreased the proportion of alveolar macrophages in both HFD and RCD mice (Fig. 4a). We also observed that RCD mice had a significantly higher proportion of interstitial macrophages with LPS challenge but this was not observed in HFD mice (Fig. 4b).

### Differential expressions of CD86 and CD206 in lung cell populations

**Figure 5:**
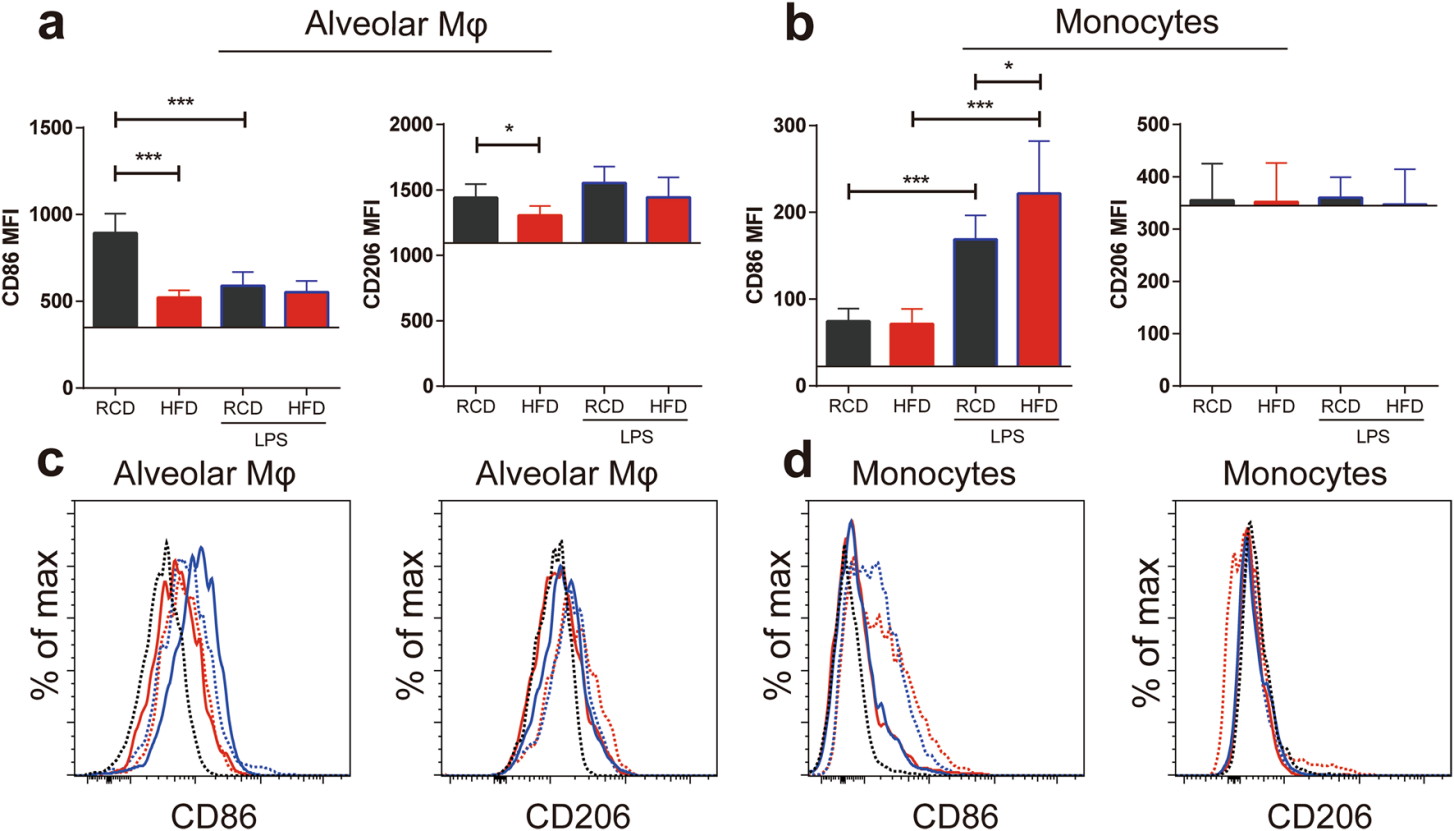
CD86 and CD206 expression levels in Cell Populations in Obesity. (**a**, **b**) CD86 and CD206 expressions in alveolar macrophages and monocytes with or without LPS challenge. Obesity reduced the expression levels of CD86 and CD206 in alveolar macrophages, but this change was not present in monocytes. CD86 expressions in alveolar macrophages were decreased in RCD mice after the LPS challenge (**a**). CD86 expressions in monocytes were increased in RCD and HFD mice after LPS challenge (**b**). Detailed statistical results can be seen in supplementary Table S7. RCD, n = 8; RCD + LPS, n = 8; HFD, n = 8; HFD + LPS, n = 10. The horizontal line in each graph was the background MFI compared with isotype control. CD86 MFI in alveolar macrophages: *P* < 0.001, HFD group compared to RCD group; CD206 MFI in alveolar macrophages:* P* < 0.05, comparing HFD group to RCD group; CD86 MFI in monocytes: *P* < 0.001, RCD + LPS group vs RCD group; *P* < 0.001, HFD + LPS group vs HFD group; *P* < 0.05, HFD + LPS group compared to RCD + LPS group; (**c**, **d**): histogram of CD86, CD206 MFI of alveolar macrophages, monocytes in RCD (blue line) mice, RCD + LPS (blue dash line) mice, HFD (red line) mice, HFD + LPS (red dash line) mice. Isotype control, black dash line. RCD, regular chow diet. HFD, high-fat diet. LPS, lipopolysaccharide. MFI, median fluorescence intensity. Data were presented as Mean ± SD. ***P < 0.001; **P < 0.01; *P < 0.05.

We further analyzed CD86 and CD206 expressions in alveolar macrophages and monocytes. We observed that the expressions of CD86 in alveolar macrophages were decreased in HFD mice as compared to RCD mice (Fig. 5a). Similar results were observed in the expressions of CD206 in alveolar macrophages (Fig. 5a). Monocytes expressed lower levels of CD86 than alveolar macrophages (Fig. 5b). There was no detectable CD206 in monocytes as compared to isotype controls (Fig. 5b). Following LPS treatment, the expressions of CD86 in macrophages were not significantly different between HFD and RCD mice. However, the expressions of CD86 were decreased in alveolar macrophages in RCD mice (Fig. 5a). The expressions of CD86 in monocytes were increased after LPS treatment (Fig. 5b) and the expressions of CD86 in monocytes were higher in HFD mice than RCD mice after LPS challenge (Fig. 5b).

### Changes in α7nAChR-positive lung cell populations in HFD mice

**Figure 6:**
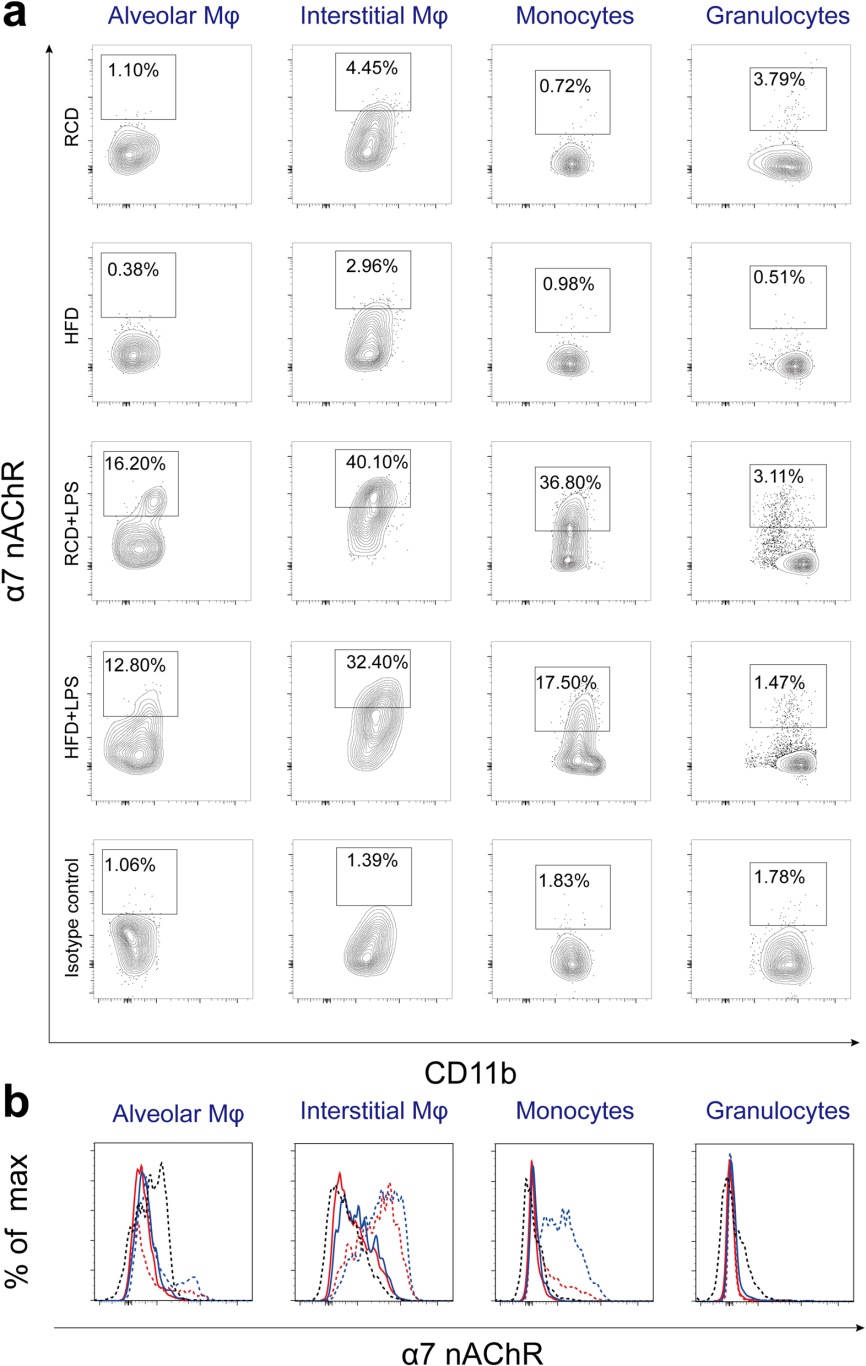
Identification of α7nAChR positive lung cell populations and gating out α7nAChR^+^ populations in alveolar macrophages, interstitial macrophages, monocytes, granulocytes. (**a**) The percentage of α7nAChR^+^ cell in four inflammatory cell subsets. Leukocytes were analyzed by FACS to quantify leukocyte subsets and their expression levels of α7nAChR^+^. Gate in each graph indicates α7nAChR^+^ populations. (**b**) Histogram of α7nAChR expression levels of alveolar macrophages, interstitial macrophages, monocytes and granulocytes. RCD group, blue solid line; HFD group, red solid line; RCD + LPS group, blue dash line; HFD + LPS group, red dash line. Isotype control, black dash line. RCD, regular chow diet. HFD, high-fat diet. LPS, lipopolysaccharide.

**Figure 7:**
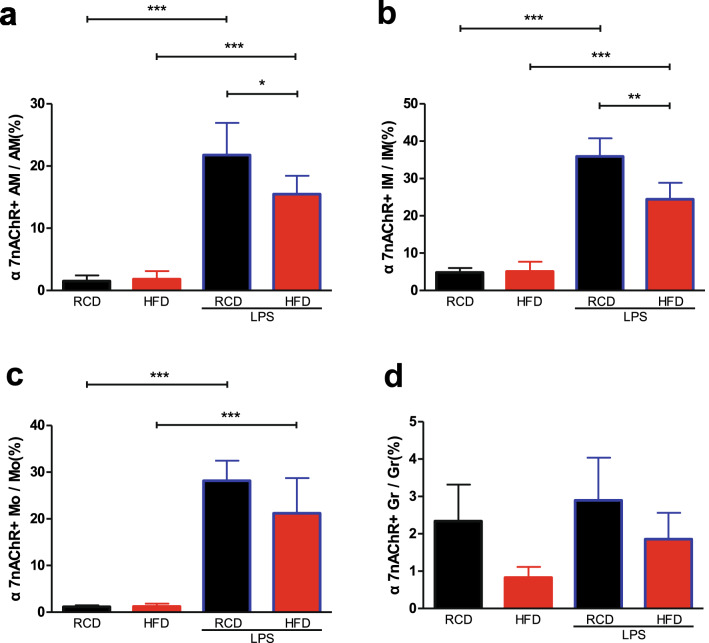
Changes in α7nAChR-expressing lung cell populations in obese mice. The ratio of α7nAChR^+^ interstitial macrophages (**b**), α7nAChR^+^ monocytes (**c**) and α7nAChR^+^ alveolar macrophages (**a**) were increased after stimulation of LPS. Detailed statistical results can be seen in Supplementary Table S8. RCD group, n = 8; RCD + LPS group, n = 8; HFD group, n = 8; HFD + LPS group, n = 10. α7nAChR^+^ AM/AM: *P* < 0.001, comparing RCD + LPS group to RCD group; *P* < 0.001, comparing HFD + LPS group to HFD group; *P* < 0.05, comparing HFD + LPS group to RCD + LPS group; α7nAChR^+^ IM/IM: *P* < 0.001, comparing RCD + LPS group to RCD group; *P* < 0.001, comparing HFD + LPS group to HFD group; *P* < 0.01, comparing HFD + LPS group to RCD + LPS group. α7nAChR^+^ Mo/Mo: *P* < 0.001, comparing RCD + LPS group to RCD group; *P* < 0.001, comparing HFD + LPS group to HFD group. RCD, regular chow diet. HFD, high-fat diet. LPS, lipopolysaccharide. IM, interstitial macrophages. Mo, monocytes. AM, alveolar macrophages. Gr, Granulocytes. Data were presented as Mean ± SD. ***P < 0.001; **P < 0.01; *P < 0.05 by Welch’s ANOVA, post-hoc Games–Howell method.

We next examined the percentages of α7nAChR-positive cells in four types of inflammatory cells in the lung (Fig. 6). We observed that only a small proportion of interstitial macrophages expressed α7nAChR (Fig. 7b). Interestingly, following LPS treatment the expressions of α7nAChR were increased in alveolar macrophages (Fig. 7a), interstitial macrophages (Fig. 7b) and monocytes (Fig. 7c), but not detectable in granulocytes (Fig. 7d) in RCD and HFD mice. Further investigation showed that the proportion of α7nAChR^+^ cells in both alveolar and interstitial macrophages were lower in HFD mice than RCD mice after the LPS challenge (Fig. 7a,b).

**Figure 8:**
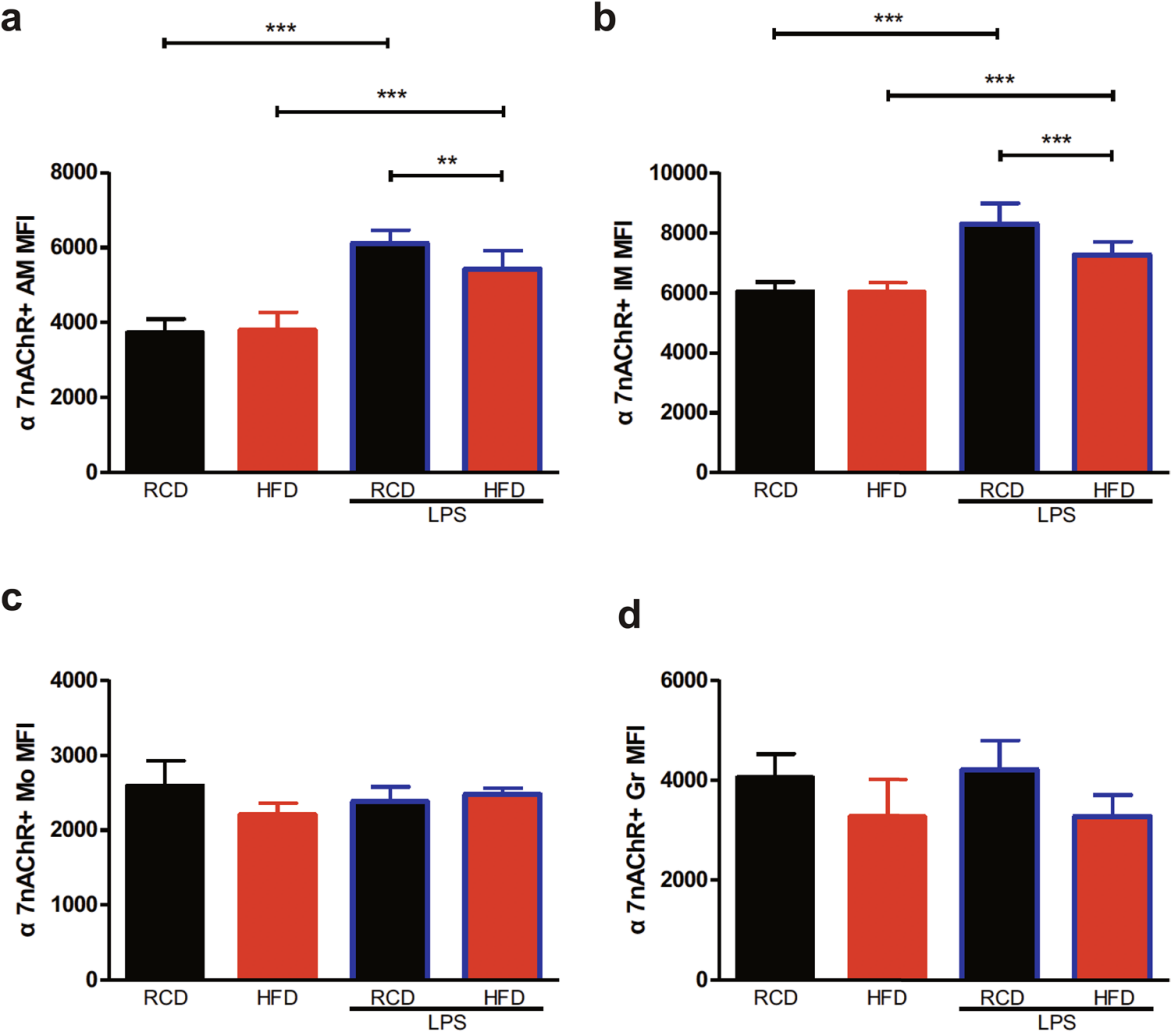
Changes of α7nAChR expression levels in Lung Cell Populations in Obese Mice. The α7nAChR expression levels of α7nAChR^+^ alveolar macrophages (**a**), α7nAChR^+^ interstitial macrophages (**b**) were increased after stimulation of LPS. Detailed statistical results can be seen in Supplementary Table S9. RCD group, n = 8; RCD + LPS group, n = 8; HFD group, n = 8; HFD + LPS group, n = 10. α7nAChR^+^ AM MFI: by ANOVA, post-hoc LSD-t method. *P* < 0.001, comparing RCD + LPS group to RCD group; *P* < 0.001, comparing HFD + LPS group to HFD group; *P* < 0.01, comparing HFD + LPS group to RCD + LPS group. α7nAChR^+^ IM MFI: by ANOVA after logarithmic transformation, post-hoc LSD-t method. *P* < 0.001, comparing RCD + LPS group to RCD group; *P* < 0.001, comparing HFD + LPS group to HFD group; *P* < 0.001, comparing HFD + LPS group to RCD + LPS group. RCD, regular chow diet. HFD, high-fat diet. LPS, lipopolysaccharide. IM, interstitial macrophages. Mo, monocytes. AM, alveolar macrophages. Gr, Granulocytes. MFI, median fluorescence intensity. Data were presented as Mean ± SD. ***P < 0.001; **P < 0.01.

Meanwhile, we analyzed the changes in the expression levels of α7nAChR. Following the LPS treatment, we observed that the expression levels of α7nAChR in both alveolar and interstitial macrophages were increased in both HFD and RCD mice (Fig. 8a,b). Unexpectedly, the expression levels of α7nAChR in lung interstitial macrophages were significantly lower in HFD mice than RCD mice following the LPS stimulation (Fig. 8b), and similar effects were observed in alveolar macrophages (Fig. 8a). No significant differences were observed in α7nAChR expressions in other populations (Fig. 8c,d).

## Discussion

In this study, we identified the cell populations expressing α7nAChR involved in the obesity-induced lung inflammation and characterized differential changes of α7nAChR expressions in obesity-induced lung inflammation with or without LPS challenges.

In the present study, we observed more lipid droplets accumulated in the lung in HFD mice, consistent with previous studies^37^. Further investigation demonstrated that some of the lipid deposits were located in macrophages. The interactions of lipid metabolism and macrophages have been investigated in previous studies^38^. We also observed that there were more alveolar macrophages in HFD mice than RCD mice, suggesting that alveolar macrophages might partake in the regulations of the increased lipid in the lung. Meanwhile, we noticed that the amount of lipid droplets decreased in HFD mice after LPS challenge, which might be attributable to increased lipolysis^39^.

CD206 was identified as a marker of M2 activation^40^ and CD86 was used as a marker of M1 activation of macrophages^41^. Recent studies demonstrated that macrophages polarized towards a mixed phenotype in adipose tissues in mice fed a high-fat diet^41,42^. CD86 (also known as B7-2) plays an important role in providing co-stimulatory signals required for T-cell activation and survival^43^. The expressions of CD86 were reduced in adipose tissue macrophages in HFD mice, which in turn causes more inflammatory macrophages infiltration^44^. Consistent with previous studies in adipose tissue^41,45^, we observed that the expression levels of CD86 in alveolar macrophages were decreased in HFD mice lungs as compared to RCD mice, which might contribute to the enhanced inflammatory status in HFD mice. We observed that LPS challenge decreased CD86 expressions in alveolar macrophages in RCD mice, which is in accordance with previous study^46^. Identical to previous study^47^, the expressions of CD86 in monocytes were increased after LPS challenge in RCD and HFD mice.

The CAP plays an important role in the regulation of obesity-induced inflammation and metabolic disorders. α7nAChR agonist or acetylcholinesterase inhibitor acting on this pathway alleviates obesity-related inflammation and metabolic complications. Previous studies have shown the infiltration of M1 type macrophage was enhanced in adipose tissue in α7nAChR knockout mice^48^. Literature shows that approximately 20% of CD45^+^ cells expressed α7nAChR in the lung^49^. In contrast to CD45^+^, CD11b^+^, CD8^+^ and B220^+^ cells, F4/80^+^, Gr1^+^ and CD11c^+^ cells exhibited different expression levels of α7nAChR^50,51^. These cells were able to migrate from the blood following LPS challenge^52^. Contrasting to previous studies^49^, we found that only a small fraction of interstitial macrophages but not alveolar macrophages expressed α7nAChR in normal conditions. Meanwhile, a study by Mikulski W et.al showed that no α7nAChR mRNA expression was observed in bronchoalveolar lavage cells^53^, and we did not observe either.

In accordance to previous studies^54^, following LPS challenge the proportion of α7nAChR^+^CD11b^+^ cells were greatly increased, which might be recruited from spleen or bone marrows. However, our multicolor flow cytometry studies revealed that the main fraction of these α7nAChR^+^ cells were alveolar and interstitial macrophages and monocytes. Although the α7nAChR^+^ cell proportions in both alveolar and interstitial macrophages were increased in HFD and RCD mice, we found that the proportion of α7nAChR^+^ cells in HFD mice was lower than RCD mice following LPS challenge, suggesting that the CAP pathway was probably compromised in anti-inflammation.

We also found that α7nAChR^+^ cells did not exist in granulocytes, suggesting that α7nAChR^+^ granulocytes may not play a major role in lung inflammatory response. Giebelen et al*.*^26^ explored the relationship between α7nAChR and lung inflammation and reported that local administration of GTS-21 reduced the levels of TNF-α in the respiratory airways of mice following LPS stimulation, whereas GTS-21 did not affect neutrophil exudation in the mouse alveolar tissues. These results indicate that the neutrophils were not probably involved in the α7 nAChR-mediated anti-inflammatory effects.

Cancello et al. found that α7nAChR expression was significantly lower in human subcutaneous adipose tissue of obese subjects compared with that of normal-weight subjects^55^. The expression levels of α7nAChR in monocytes and alveolar and interstitial macrophages were all increased following LPS stimulation, suggesting that they were probably involved in the cholinergic pathway-mediated inflammatory response in the lung. It is worth noting that the α7nAChR expression levels were significantly lower in α7nAChR^+^ interstitial macrophages following LPS stimulation in HFD mice than RCD mice in our study, indicating that obesity affected α7nAChR expressions in lung interstitial macrophages after LPS challenge. This study further indicated the decreased expression levels of α7nAChR in lung interstitial macrophages may alter the regulation of CAP pathway on inflammatory response in obesity treated with LPS.

Meanwhile, it is well known that the interstitial macrophages are closely associated with interstitial T lymphocytes and dendritic cells in the lungs and that they play an important role in the development of inflammation and other immune functions^56^. Therefore, lung interstitial macrophages are likely to be involved in the construction of vagal anti-inflammatory pathways in the lung. Liegeois et al*.*^57^ pointed out that a percentage of the lung interstitial macrophages could be supplemented by the monocytes in the blood circulation, we presumed that α7nAChR^+^ interstitial macrophages may be derived from α7nAChR^+^ monocytes, which probably recruited from the spleen after LPS challenge^54^.

Collectively, the present study demonstrates that the α7nAChR are predominantly expressed in interstitial and alveolar macrophages and monocytes in mouse lungs, and obesity alters the proportion of α7nAChR^+^ cells in alveolar and interstitial macrophages and the expression levels of α7nAChR upon the LPS challenge, which may have an impact on the cholinergic anti-inflammatory pathway, providing a potential therapeutic target for clinical neurogenic immuno-anti-inflammatory treatment.

## Supplementary information

Supplementary Information
