# Supplementary material for: High-fat diet-induced obesity affects alpha 7 nicotine acetylcholine receptor expressions in mouse lung myeloid cells
Source: Sci Rep. 2020 Oct 27;10:18368. doi: 10.1038/s41598-020-75414-w (PMC7592050; doi:10.1038/s41598-020-75414-w)
Supplement: Supplementary file 1 — Supplementary Information [file 41598_2020_75414_MOESM1_ESM.pdf]

High-Fat Diet-Induced Obesity Affects Alpha 7 Nicotine Acetylcholine Receptor Expressions in Mouse Lung Myeloid Cells

Yong Qi[1*], Dan Si[1,2], Li Zhu[1], Yanan Qi[1], Zhuhua Wu[1], Dan Chen[1,3], & Yunlei Yang[3,4,5,6*]

Supplementary information including:

2 figure legends;

2 figures;

9 tables;

Statistical justification around sample size selection.

Supplementary Figure Legends

Supplementary Figure 1. A time line of the experimental procedures.

Supplementary Figure 2. Sequence gating strategy for cell identifications. Cells were isolated from enzymatically digested mouse lungs and, after exclusion of doublets and debris, leukocytes were identified by $CD45^+$ staining. Sequential gating strategy was applied to identify populations expressing specific markers. Alveolar macrophages express lower levels of CD24 compared to CD103+DCs[1] CD64 is a marker which can discriminate monocyte-derived DCs and alveolar macrophage from conventional DCs. CD11b-CD11c+ cells doesn't comprise monocyte-derived DCs, as monocyte-derived DCs are CD11b+CD11c+MHCII+ cells[2].Alveolar macrophages were identified as $CD11b^-CD11c^+$ cells, further purified by using CD64 and CD24 and the CD24+CD64- cells were CD103+DCs[3]. Gating on $CD11b^{hi}$ cells allows for the separation of myeloid cells from lymphoid cells that either do not express this marker (T and B cells), or express it at intermediate level (natural killer cells). Granulocytes (neutrophils and eosinophils) can be gated out as $CD24^+CD11c^-$, and the identification of interstitial macrophages ($CD11c^+CD11b^+$MHC $II^+CD24^-CD64^+$), and monocytes can be continued as in the full panel ($CD11b^+$MHC $II^-CD64^{+/-}$). In a simplified panel, almost all alveolar macrophages were $CD64^+$, so alveolar macrophages can be classified as $CD11b^-CD11c^+CD24^{low}$. Nearly all monocytes were MHC II^-, so monocytes can be classified as MHC II^-, regardless of the expression level of CD64. FSC, forward scatter; MHC II, major histocompatibility complex class II; SSC, side scatter.

1 Becher, B. et al. High-dimensional analysis of the murine myeloid cell system. Nat. Immunol. 15, 1181-1189, (2014).

2 Guilliams, M., Lambrecht, B. N. & Hammad, H. Division of labor between lung dendritic cells and macrophages in the defense against pulmonary infections. Mucosal Immunol. 6, 464-473, (2013).

3 Misharin, A. V., Morales-Nebreda, L., Mutlu, G. M., Budinger, G. R. & Perlman, H. Flow cytometric analysis of macrophages and dendritic cell subsets in the mouse lung. Am. J. Respir. Cell Mol. Biol. 49, 503-510, (2013).

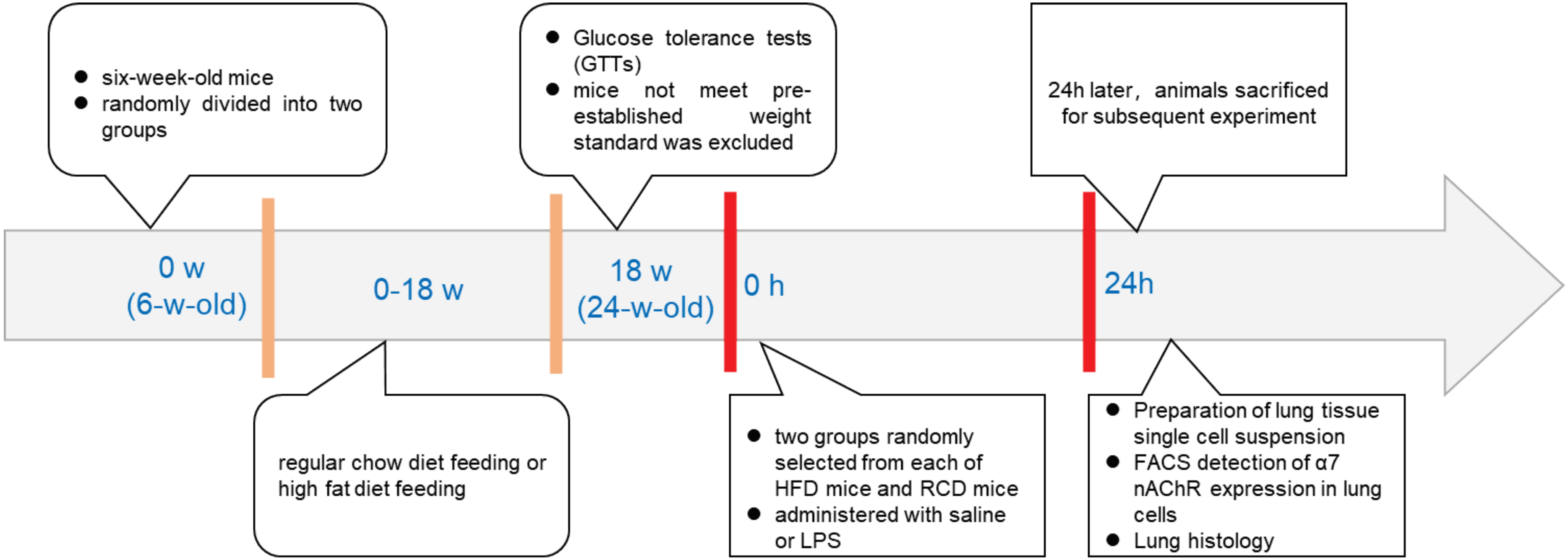
six-week-old mice
randomly divided into two groups
Glucose tolerance tests (GTTs)
mice not meet pre-established weight standard was excluded
24h later, animals sacrificed for subsequent experiment
0 w (6-w-old)
0-18 w
18 w (24-w-old)
0 h
24h
regular chow diet feeding or high fat diet feeding
two groups randomly selected from each of HFD mice and RCD mice
administered with saline or LPS
Preparation of lung tissue single cell suspension
FACS detection of α7 nAChR expression in lung cells
Lung histology

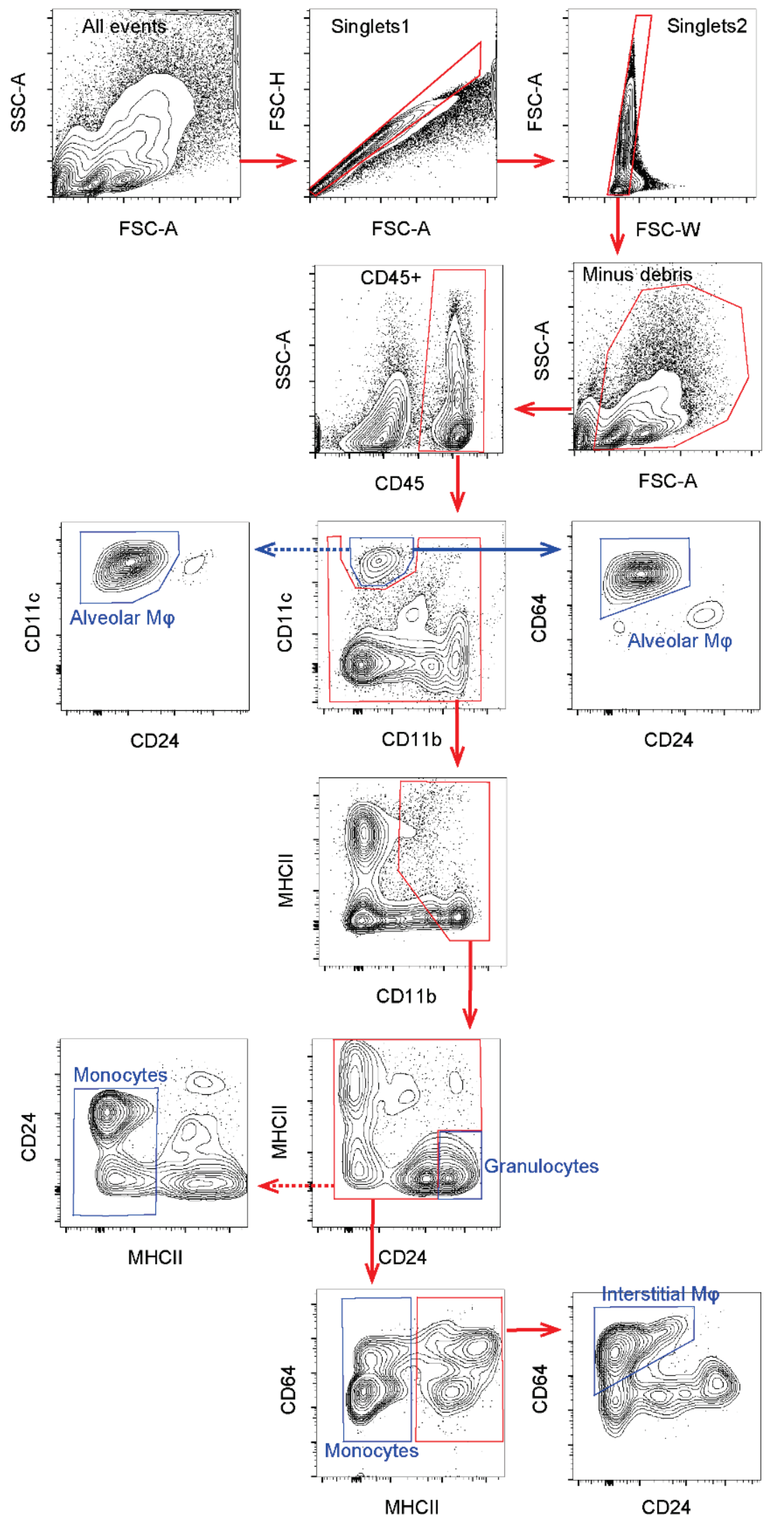

All events
SSC-A
FSC-A
Singlets1
FSC-H
FSC-A
Singlets2
FSC-A
FSC-W
CD45+
SSC-A
CD45
Minus debris
SSC-A
FSC-A
CD11c
CD24
Alveolar Mφ
CD11c
CD11b
CD64
CD24
Alveolar Mφ
MHCII
CD11b
Monocytes
CD24
MHCII
MHCII
CD24
Granulocytes
CD64
MHCII
Monocytes
Interstitial Mφ
CD64
CD24

Supplementary Table S1 : The nutrient content of regular chow diet.

Regular chow diet	Water	Crude protein	Crude fat	Carbohydrate	Crude fiber	Crude ash	Calcium	Total phosphorus	Vitamins	Minerals & Minor elements	Required amino acid
	10%	22.5%	4.20%	51%	3.27%	6.24%	1.25%	0.77%	⩽0.3%	⩽0.5%	⩾5.00%

Caloric Information: Protein, 27 % Kcal; Fat, 11 % Kcal; Carbohydrate, 61 % Kcal; Energy density, 3.32 Kcal/g.

Supplementary Table S2 : The ingredients of high-fat diet.

High-fat diet	Casein, Lactic, 30 Mesh	Cystine, L	Lodex 10	Sucrose Fine Granulated	Solka floc, FCC 200	Lard	Soybean Oil, USP	S10026B	Choline Bitartrate	V10001C	Dye, Blue FD&C #1, Alum. Lake 35-42 %
	200 g	3.0 g	125.0 g	72.8 g	50.0 g	245.0 g	25.0 g	50.0 g	2.0 g	1.0 g	0.05 g
	26.8 %	0.4 %	16.2 %	9.4 %	6.5 %	31.7 %	32.3 %	6.5 %	0.3 %	0.1 %	＜ 0.01 %

Caloric Information: Protein, 20 % Kcal; Fat, 60 % Kcal; Carbohydrate, 20 % Kcal; Energy density, 5.21 Kcal/g.
V10001C is an AIN-76A vitamin mix (10X concentration) additive according to Research Diets, Inc. The detailed ingredient of it can be found in https://researchdiets.com/formulas/V10001C.

Antigen	Clone	Fluorochrome	Dilution	Manufacturer
CD11b	M1/70	APC-Cy7	2.5:100	Biolegend
CD11c	N418	PE-Cy7	1.25:100	Biolegend
MHC-II	2G9	BB515	1.25:100	BD Bioscience
CD24	M1/69	PE-CF594	2.5:100	BD Bioscience
	30-F1	APC	3.75:100	Biolegend
CD64	X54-5/7.1	BV 421	1:100	Biolegend
CD86	GL-1	BV 421	1:100	Biolegend
CD45	30-F11	PerCP-Cy5.5	1:100	eBioscience
α7AChR	319	PE	5:100	Santa Cruz
CD206	MR6F3	APC	1.25:100	eBioscience
α7AChR isotype	R3-34	PE	5:100	BD Bioscience
CD86 isotype	RTK2758	BV 421	1:100	Biolegend
CD206 isotype	eB149/10H5	APC	1.25:100	eBioscience

Supplementary Table S3 : Antibodies panel used for flow cytometric analysis. APC-Cy7 is allophycocyanin-Cyanine7; PE-Cy7 is Phycoerythrin-Cyanine7; BB515 is a dye which can provide a brighter alternative to FITC with less spillover into the PE detector; PE-CF594 is a dye which has been developed as a better alternative to PE-Texas Red; APC is allophycocyanin; BV421 is Brilliant Violet 421, it is excited at 405 nm and emits at 421 nm; PerCP-Cy5.5 is Peridinin Chlorophyll-Cyanine5.5; PE is phycoerythrin.

Supplementary Table S4 : Cumulative increase of body weight (g). *** *P*<0.001, comparing HFD mice to RCD-fed mice at the same weeks. HFD, high fat diet, n=18; RCD, regular chow diet, n=16. Data was presented as Mean±SD. Normality test was determined by Shapiro-Wilk, statistical significance was determined by ANOVA for repeatedly measured data, followed by post-hoc Bonferroni method.

Weeks	6 w	8 w	10 w	12 w	14 w	16 w	18 w	20 w	22 w	24 w
RCD	21.1±0.8	23.0±1.0	24.6±1.3	25.9±1.5	27.4±0.8	28.3±1.0	28.0±2.0	28.7±1.1	28.9±1.6	30.1±1.1
HFD	20.7±0.9	25.5±1.4 ***	28.5±1.3 ***	33.8±2.1 ***	37.3±3.3 ***	40.8±3.9 ***	41.6±4.3 ***	42.5±4.4 ***	43.7±4.6 ***	46.1±4.1 ***

Supplementary Table S5 : Glucose tolerance test (GTT). *** *P*<0.001, comparing HFD group to RCD group at the same time. High fat diet, n=18; Regular chow diet, n=16. Data was presented as Mean±SD. Normality test was determined by Shapiro-Wilk, statistical significance was determined by ANOVA for repeatedly measured data, followed by post-hoc Bonferroni method.

Groups	0min	30min	60min	90min	120min
RCD	6.0±0.6	17.8±2.6	13.6±3.2	10.0±1.3	8.1±0.7
HFD	6.3±0.7	27.8±3.4 ***	26.4±3.5 ***	23.7±3.1 ***	20.2±2.5 ***

Supplementary Table S6 : The percentage of leukocytes, alveolar macrophages, interstitial macrophages, monocytes, granulocytes in the total cell count. RCD, n=8; RCD+LPS, n=8; HFD, n=8; HFD+LPS, n=10. *** *P*<0.001, ** *P* <0.01, * *P* <0.05 , comparing HFD group to RCD group; ### *P*<0.001, ## *P* <0.01, # *P* <0.05 , comparing RCD+LPS group to RCD group or comparing HFD+LPS group to HFD group; †††*P*<0.001, ††*P*<0.01, †*P*<0.05 , comparing HFD+LPS group to RCD+LPS group. RCD, regular chow diet. HFD, high fat diet. LPS, lipopolysaccharide. Data were presented as Mean±SD. Normality test was determined by Shapiro-Wilk, Alveolar macrophages / Total (Minus Debris) (%): by ANOVA, post-hoc LSD-t method. Interstitial macrophages / Total (Minus Debris) (%): by ANOVA after logarithmic transformation, post-hoc LSD-t method. Monocytes / Total (Minus Debris) (%): by ANOVA after logarithmic transformation, post-hoc LSD-t method. Granulocytes / Total (Minus Debris) (%): by Welch's ANOVA, post-hoc Games-Howell method.

Groups	**leukocytes(CD45+) / Total(Minus Debris)**	**Alveolar macrophages / Total(Minus Debris)**	**Interstitial macrophages / Total(Minus Debris)**	**Monocytes / Total(Minus Debris)**	**Granulocytes / Total(Minus Debris)**
RCD	36.20±3.40	0.66±0.10	1.27±0.11	1.01±0.21	1.71±0.59
HFD	43.51±2.41 ***	1.05±0.13 ***	1.64±0.53	1.38±0.61	3.09±2.04
RCD+LPS	52.20±3.78 ###	0.46±0.27 #	2.06±0.41 ###	5.49±1.51 ###	17.38±4.35 ###
HFD+LPS	66.31±6.85 ### †††	0.35±0.19 ###	1.98±0.35	8.91±1.83 ### ††	19.97±4.44 ###

Supplementary Table S7 : The expression levels of CD86, CD206 in alveolar macrophages and monocytes. RCD, n=8; RCD+LPS, n=8; HFD, n=8; HFD+LPS, n=10. *** *P*<0.001, ** *P* <0.01, * *P* <0.05 , comparing HFD group to RCD group; ### *P*<0.001, ## *P* <0.01, # *P* <0.05 , comparing RCD+LPS group to RCD group or comparing HFD+LPS group to HFD group; †††*P*<0.001, ††*P*<0.01, †*P*<0.05 , comparing HFD+LPS group to RCD+LPS group. RCD, regular chow diet. HFD, high fat diet. LPS, lipopolysaccharide. Mo, monocytes; AM, alveolar macrophages. Data was presented as Mean±SD. Normality test was determined by Shapiro-Wilk, CD86 MFI (AM): by ANOVA after logarithmic transformation, post-hoc LSD-t method. CD206 MFI (AM): by Welch's ANOVA, post-hoc Games-Howell method. CD86 MFI (Mo): by ANOVA, post-hoc LSD-t method.

Groups	CD86 MFI (AM)	CD206 MFI (AM)	CD86 MFI (Mo)	CD206 MFI (Mo)
RCD	894±111	1440±104	74±15	355±70
HFD	523±40 ***	1305±74 *	71±17	352±74
RCD+LPS	590±79 ###	1553±126	169±28 ###	245±36
HFD+LPS	553±64	1444±153	221±60 ### †	281±58

Supplementary Table S8 : The percentage of α7nAChR^{+} cells in four inflammatory cell subsets. RCD, n=8; RCD+LPS, n=8; HFD, n=8; HFD+LPS, n=10. ### *P*<0.001, ## *P* <0.01, #p<0.05 , comparing RCD+LPS group to RCD group or comparing HFD+LPS group to HFD group; †††*P*<0.001, ††*P*<0.01, †*P*<0.05 , comparing HFD+LPS group to RCD+LPS group. RCD, regular chow diet. HFD, high fat diet. LPS, lipopolysaccharide. AM, alveolar macrophages. IM, interstitial macrophages. Mo, monocytes. Gr, granulocytes. Data were presented as Mean±SD. Normality test was determined by Shapiro-Wilk, statistical significance was determined by Welch's ANOVA, post-hoc Games-Howell method.

Groups	α7nAChR^{+} AM / AM	α7nAChR^{+} IM / IM	α7nAChR^{+} Mo / Mo	α7nAChR^{+} Gr / Gr
RCD	1.51±0.91	4.85±1.18	1.15±0.33	2.34±0.98
HFD	1.84±1.28	5.11±2.60	1.24±0.61	0.83±0.28
RCD+LPS	21.76±5.17 ###	35.88±4.88 ###	28.18±4.31 ###	2.90±1.14
HFD+LPS	15.50±2.93 ### †	24.42±4.44 ### ††	21.18±7.57 ###	1.86±0.71

Supplementary Table S9 : The expression levels of α7nAChR in each cell subpopulations. RCD, n=8; RCD+LPS, n=8; HFD, n=8; HFD+LPS, n=10. comparing HFD group to RCD group; ### P<0.001, ## P<0.01, # P<0.05 , comparing RCD+LPS group to RCD group or comparing HFD+LPS group to HFD group; †††P<0.001, ††P<0.01, †P<0.05 , comparing HFD+LPS group to RCD+LPS group. RCD, regular chow diet. HFD, high fat diet. LPS, lipopolysaccharide. AM, alveolar macrophages. IM, interstitial macrophages. Mo, monocytes. Gr, granulocytes. Data were presented as Mean±SD. Normality test was determined by Shapiro-Wilk, α7nAChR+ AM MFI: by ANOVA, post-hoc LSD-t method. α7nAChR+ IM MFI: by ANOVA after logarithmic transformation, post-hoc LSD-t method.

Groups	α7nAChR^{+} AM MFI	α7nAChR^{+} IM MFI	α7nAChR^{+} Mo MFI	α7nAChR^{+} Gr MFI
RCD	3723±376	6023±330	2598±491	4047±474
HFD	3799±475	6015±328	2232±316	3273±756
RCD+LPS	6107±365 ###	8288±695 ###	2431±176	4213±589
HFD+LPS	5431±492 ### ††	7251±450 ### †††	2476±104	3279±429

Statistical justification around sample size selection

In our ANOVA model, we performed the power analysis via SPSS and G*Power software[1,2] post hoc. The results were listed in the following tables. According to previous methods[1], the effect size in ANOVA model, Cohen's f has a relationship with partial η^2, $f = \sqrt{\frac{\eta 2}{1-\eta 2}}$. Following pre-established standard[1]， small effect size is f=0.10, η^2=0.01; medium effect size is f=0.25, η^2=0.06; large effect size is f=0.40, η^2=0.14.

	cd45/minus debris	alveolar macrophages/ minus debris	interstitial macrophages/ minus debris	monocyte/min us debris	granulocytes /minus debris
sig.level	0.05	0.05	0.05	0.05	0.05
η^2	0.876	0.705	0.460	0.924	0.914
power	1.000	1.000	0.986	1.000	1.000

	alveolar macrophages α7+ / alveolar macrophages	interstitial macrophages α7+/ interstitial macrophages	monocytes α7+ / monocytes	granulocytes α7+ / granulocytes
sig.level	0.05	0.05	0.05	-
η^2	0.853	0.918	0.939	-
power	1.000	1.000	1.000	-

	alveolar macrophages α7+ MFI	interstitial macrophages α7+ MFI	monocytes α7+ MFI	granulocytes α7+ MFI
sig.level	0.05	0.05	0.05	-
η^2	0.860	0.812	0.171	-
power	1.000	1.000	0.475	-

After calculation, we found that under the current sample size (n=34), the test power of the α7 expression intensity of monocytes did not reach 0.8, and the test power of other groups all met the requirements (> 0.8). When calculated according to our second lowest effect size η2 = 0.460, Cohen's f = 0.92. Then we calculated via G*Power software to evaluate the required sample size, by giving significant level = 0.05, 4 groups, and test power 0.8, the required sample size is 5 per group. And to our lowest effect size η2 = 0.171 (the test power of the α7 expression intensity of monocytes), Cohen's f = 0.50. The calculated required sample size, by giving significant level = 0.05, 4 groups, and test power 0.8, is 13 per groups. So, except the α7 expression intensity of monocytes, our sample size was able to test the difference between groups.

1 Cohen, J. Statistical power analysis for the behavioral sciences. (Academic press, 2013).
2 Faul, F., Erdfelder, E., Lang, A. G. & Buchner, A. G*Power 3: a flexible statistical power analysis program for the social, behavioral, and biomedical sciences. Behav Res Methods 39, 175-191, doi:10.3758/bf03193146 (2007).
